# Converting health risks into loss of life years - a paradigm shift in clinical risk communication

**DOI:** 10.18632/aging.203491

**Published:** 2021-09-07

**Authors:** Shan Pou Tsai, Chi Pang Wen, Min Kuang Tsai, Po Jung Lu, Jackson Pui Man Wai, Christopher Wen, Wayne Gao, Xifeng Wu

**Affiliations:** 1 MJ Health Management Institution, Taipei, Taiwan; 2 Institute of Population Health Science, National Health Research Institutes, Zhunan, Taiwan; 3 China Medical University Hospital, Taichung, Taiwan; 4 Institute of Sport Science, National Taiwan Sport University, Taoyuan, Taiwan; 5 Long Beach VAMC Hospital, University of Irvine Medical Center, Irvine, CA 92868, USA; 6 Taipei Medical University, Taipei, Taiwan; 7 Center for Biostatistics, Bioinformatics and Big Data, The Second Affiliated Hospital and School of Public Health, Zhejiang University School of Medicine, Hangzhou, China; 8 National Institute for Data Science in Health and Medicine, Zhejiang University, Hangzhou, China

**Keywords:** life expectancy, health risks, hazard ratio, mortality, cohort

## Abstract

For facilitating risk communication in clinical management, such a ratio-based measure becomes easier to understand if expressed as a loss of life expectancy. The cohort, consisting of 543,410 adults in Taiwan, was recruited between 1994 and 2008. Health risks included lifestyle, biomarkers, and chronic diseases. A total of 18,747 deaths were identified. The Chiang’s life table method was used to estimate a loss of life expectancy. We used Cox regression to calculate hazard ratios (HRs) for health risks. The increased mortality from cardio-metabolic risks such as high cholesterol (HR=1.10), hypertension (HR=1.48) or diabetes (HR=2.02) can be converted into a loss of 1.0, 4.4, and 8.9 years in life expectancy, respectively. The top 20 of the 30 risks were associated with a loss of 4 to 10 years of life expectancy, with 70% of the cohort having at least two such risk factors. Smoking, drinking, and physical inactivity each had 5-7 years loss. Individuals with diabetes or an elevated white count had a loss of 7-10 years, while prolonged sitting, the most prevalent risk factor, had a loss of 2-4 years. Those with diabetes (8.9 years) and proteinuria (9.1 years) present at the same time showed a loss of 16.2 years, a number close to the sum of each risk. Health risks, expressed as life expectancy loss, could facilitate risk communication. The paradigm shift in expressing risk intensity can help set public health priorities scientifically to promote a focus on the most important ones in primary care.

## INTRODUCTION

Much of our efforts in primary care are to identify health risks and to reduce their health impact. Thus, understanding, quantifying, and managing health risks have become one of the most important activities in clinical settings, particularly effective communication of these risks to the lay person. Currently, “relative risk” has been the most commonly used expression. The size of relative risks reported in different studies was perceived as the degree of harmfulness. Relative risk, a ratio-based measure, is not only difficult for the public to understand, but also cannot be directly compared with other relative risks, or with different reference groups. In contrast, life expectancy, derived from collapsing age-specific mortality, is an absolute risk with implications well understood to most people. Life expectancy is the average number of years a cohort of people is expected to live, which has intuitive meaning and can be compared [1, 2]. Prolonging one’s life is an overarching goal of the public health community [3] Overcoming the loss of life expectancy, on the other hand, is a clinical goal shared by everyone. Loss of life expectancy in years can be a universal yardstick across different disciplines in clinical practice, reflecting the severity of a given risk. Nevertheless, life expectancy has not been extensively used in cohort studies, and its potential has not been fully recognized, mainly because its calculation requires a large cohort with an extended follow-up time yielding stable results with a sufficient number of deaths in each age group [4–8].

In contrast to methods adopted by the Global Burden of Disease Study [9], or the study of potential years of life loss (PYLL) at a population level [10], results possibly are relevant for individuals in their daily life have been limited. Priorities based on global or societal issues are important but different, and not perceived by individuals as motivating behavioral changes. The literature seldom applies PYLL to quantify the impact of risk factors at a personal level. In addition, identified morbidities in the population are not necessarily the same as causes of death, as registered. As the reduction of health risks to extend life expectancy is among the most important clinical and public health goals, the loss of life expectancy is easy to understand and intuitively memorable. More importantly, direct comparison between risks is commonly done using life expectancy.

In this study, 30 health risks were identified from a standard medical screening program on half a million participants consecutively recruited between 1994 and 2008 in Taiwan. These risks represented common behavioral risk factors and medically screened risks. The objective of this study is to quantify the negative impact of each of these health risks on life expectancy. Loss of life expectancy was the difference in life expectancy between those with the risk and those without the risk. Years of life lost for individuals with two or three risks were also assessed against those without either one of the risks.

## RESULTS

The study population consisted of 543,410 adults, including 48% men and 52% women, with an average follow-up of 8.1 years. A total of 18,747 deaths were identified. Approximately a third of the study population was middle-aged (40-59 years) at the beginning of follow-up. The elderly (60 years and older) accounted for 13% of the cohort but over 60% of the deaths (Table 1). The magnitude and pattern of hazard ratios between univariate and multivariate were largely similar for each of the 30 risks. (Supplementary Table 1).

**Table 1:** Distribution of cohort by age, gender, and age of deaths.

**Age (years)**	**Men**			**Women**			**Total**	
	**N**	**%**		**N**	**%**		**N**	**%**
**Participants**								
20-39	142,867	55.2%		153,168	53.8%		296,035	54.5%
40-59	80,565	31.1%		95,830	33.7%		176,395	32.5%
≥ 60	35,351	13.7%		35,629	12.5%		70,980	13.1%
Total	258,783	100.0%		284,627	100.0%		543,410	100.0%
Follow-up person-year	2,048,262			2,326,632			4,374,895	
**Deaths**								
20-39	987	8.7%		599	8.0%		1,586	8.5%
40-59	3,134	27.8%		2,270	30.5%		5,404	28.8%
≥ 60	7,171	63.5%		4,586	61.5%		11,757	62.7%
Total	11,292	100.0%		7,455	100.0%		18,747	100.0%

**Figure 1:**
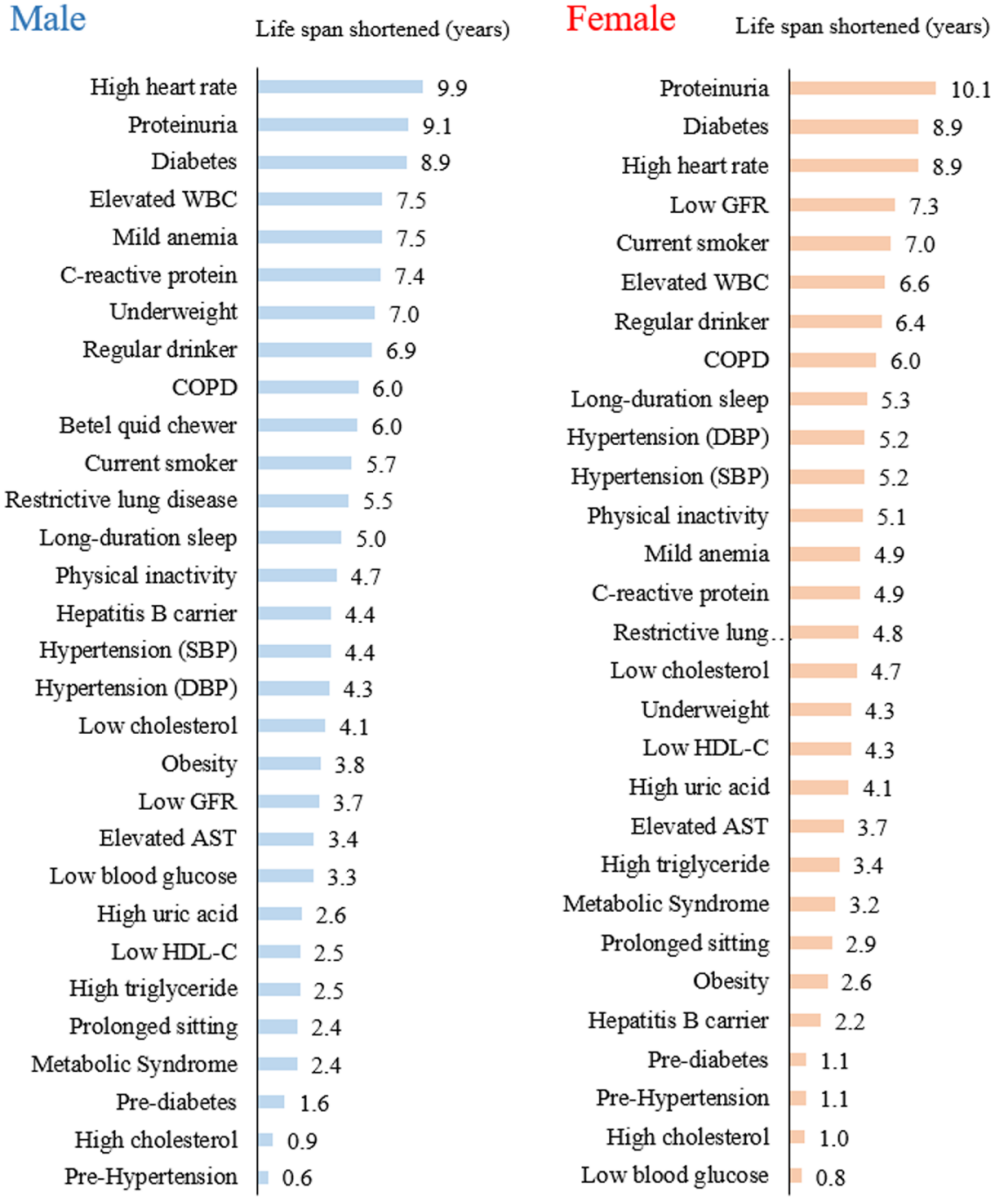
Years of life lost from each of the 30 health risks in men and women.

The top 20 of 30 risks were associated with a loss of 4 or more years of life expectancy (Table 2 and Figure 1). Six of the top 10 risks were identical between men and women with at least 6 years of life lost, and three were found to have 9 years of life shortened: high heart rate, proteinuria, and diabetes. Ninety-one percent of the cohort had at least one risk that shortened life by 4 or more years, 68% had two, and 41% had three or more (Supplementary Table 2).

**Table 2:** Prevalence and years of life lost by risk factors in men (left panel) and in women (right panel).

**Risk factor**	**Prevalence**	**HR^1^**	**Years of life lost^2^**					**Risk factor**	**Prevalence**	**HR^1^**	**Years of life lost2**			
			**Year**	**95% CI**							**Year**	**95% CI**		
High heart rate (≥ 90 beats/min)	6.4%	2.16	**9.94**	9.58	-	10.3		Proteinuria (Trace or Positive)	7.1%	2.44	**10.14**	9.93	-	10.35
Proteinuria (Trace or Positive)	7.9%	2.11	**9.07**	8.87	-	9.27		Diabetes (≥ 126 mg/dL)	4.8%	2.4	**8.91**	8.65	-	9.17
Diabetes (≥ 126 mg/dL)	5.7%	2.02	**8.92**	8.71	-	9.13		High heart rate (≥ 90 beats/min)	8.1%	2	**8.89**	8.49	-	9.29
Elevated WBC (≥ 9,000/mm^3^)	9.0%	1.82	**7.49**	7.11	-	7.87		Low GFR (< 60 ml/min/1.73 m^2^)	4.3%	1.78	**7.32**	7.1	-	7.54
Mild anemia (Hemoglobin 10 - 13.4 g/dL)	5.3%	1.56	**7.47**	7.24	-	7.7		Current smoker	6.7%	1.79	**7.02**	6.69	-	7.35
C-reactive protein (≥ 3 mg/L)	11.1%	1.84	**7.37**	7.11	-	7.63		Elevated WBC (≥ 9,000/mm^3^)	5.7%	1.9	**6.63**	6.32	-	6.94
Underweight (BMI <19 kg/m^2^)	6.3%	1.57	**7.04**	6.58	-	7.5		Regular drinker	2.4%	1.77	**6.35**	6.05	-	6.65
Regular drinker	12.1%	1.79	**6.86**	6.58	-	7.14		COPD	6.3%	1.45	**6.01**	5.76	-	6.26
COPD	7.3%	1.59	**6.04**	5.75	-	6.33		Long-duration sleep (> 8 h/day)	10.9%	1.44	**5.34**	5.02	-	5.66
Betel quid chewer	18.8%	1.82	**6.00**	5.66	-	6.34		Hypertension (DBP ≥ 90 mmHg)	17.6%	1.48	**5.21**	4.89	-	5.53
Current smoker	40.6%	1.69	**5.66**	5.35	-	5.97		Hypertension (SBP ≥ 140 mmHg)	16.4%	1.45	**5.18**	4.55	-	5.81
Restrictive lung disease	14.6%	1.55	**5.47**	5.19	-	5.75		Physical inactivity (<3.75 MET-h/wk)	59.9%	1.36	**5.07**	4.8	-	5.34
Long-duration sleep (> 8 h/day)	8.9%	1.43	**5.04**	4.75	-	5.33		Mild anemia (hemoglobin 10 - 12 g/dL)	10.2%	1.52	**4.89**	4.67	-	5.11
Physical inactivity (<3.75 MET-h/wk)	48.4%	1.55	**4.74**	4.45	-	5.03		C-reactive protein (≥ 3 mg/L)	10.5%	1.59	**4.86**	4.6	-	5.12
Hepatitis B carrier	17.4%	1.55	**4.4**	4.1	-	4.7		Restrictive lung disease	18.1%	1.45	**4.79**	4.54	-	5.04
Hypertension (SBP ≥ 140 mmHg)	19.1%	1.48	**4.39**	4.06	-	4.72		Low cholesterol (<160 mg/dL)	20.0%	1.6	**4.66**	4.21	-	5.11
Hypertension (DBP ≥ 90 mmHg)	22.0%	1.4	**4.29**	4.03	-	4.55		Underweight (BMI <18.5 kg/m^2^)	12.0%	1.46	**4.25**	3.53	-	4.97
Low cholesterol (<160 mg/dL)	17.0%	1.39	**4.06**	3.68	-	4.44		Low HDL-C (<35 mg/dL)	5.4%	1.4	**4.25**	4	-	4.5
Obesity (BMI ≥ 30 kg/m^2^)	4.3%	1.53	**3.77**	3.3	-	4.24		High uric acid (≥ 7 mg/dL)	8.5%	1.39	**4.11**	3.8	-	4.42
Low GFR (< 60 ml/min/1.73 m^2^)	6.0%	1.36	**3.67**	3.47	-	3.87		Elevated AST (≥ 25 U/L)	17.8%	1.48	**3.71**	3.5	-	3.92
Elevated AST (≥ 25 U/L)	35.9%	1.48	**3.38**	3.16	-	3.6		High triglyceride (≥ 200 mg/dL)	6.4%	1.43	**3.43**	3.15	-	3.71
Low blood glucose (< 90 mg/dL)	17.5%	1.31	**3.32**	3.06	-	3.58		Metabolic Syndrome (ATP III)	10.2%	1.43	**3.16**	2.98	-	3.34
High uric acid (≥ 8 mg/dL)	21.1%	1.23	**2.64**	2.3	-	2.98		Prolonged sitting (>8 hours/day)	61.9%	1.21	**2.92**	2.60	-	3.25
Low HDL-C (<35 mg/dL)	16.5%	1.25	**2.52**	2.26	-	2.78		Obesity (BMI ≥ 30 kg/m^2^)	3.6%	1.41	**2.61**	2.21	-	3.01
High triglyceride (≥ 200 mg/dL)	15.7%	1.21	**2.51**	2.24	-	2.78		Hepatitis B carrier	12.4%	1.35	**2.17**	1.86	-	2.48
Prolonged sitting (>8 hours/day)	55.8%	1.23	**2.37**	2.06	-	2.68		Pre-diabetes (110 - 125 mg/dL)	4.5%	1.13	**1.11**	0.85	-	1.37
Metabolic Syndrome (ATP III)	13.7%	1.26	**2.36**	2.16	-	2.56		Pre-Hypertension (SBP 120 - 139 mmHg)	21.7%	1.05	**1.09**	0.49	-	1.69
Pre-diabetes (110 - 125 mg/dL)	6.9%	1.15	**1.62**	1.4	-	1.84		High cholesterol (≥ 240 mg/dL)	10.2%	1.1	**1.03**	0.67	-	1.39
High cholesterol (≥ 240 mg/dL)	10.9%	1.13	**0.91**	0.55	-	1.27		Low blood glucose (< 90 mg/dL)	30.8%	1.13	**0.82**	0.48	-	1.16
Pre-Hypertension (SBP 120 - 139 mmHg)	36.7%	1.12	**0.60**	0.28	-	0.92								

^1^HR: Age-adjusted hazard ratio.

^2^Life years lost: those with the risk – those without the risk.

Individuals with a heart rate greater than 90 beats/minute had a substantial loss of life expectancy, 10 years for men and 9 years for women. Individuals with an elevated WBC count (>9,000/mm^3^) lost 7 years, and those with an elevated CRP (>3 mg/L) lost 5-7 years. Proteinuria, determined by dipstick, had 9-10 years loss as a whole, but a 7-year loss for those with trace proteinuria (data not shown).

In the middle range of life years lost, long-duration sleep (5 years), physical inactivity (5 years), hypertension (4-5 years), obesity (3-4 years), and prolonged sitting (2-3 years) were identified. Among the risks with minimal loss of years were high cholesterol (1 year), pre-hypertension, and pre-diabetes (1-2 years). In contrast, those with low cholesterol lost 4-5 years, while being underweight showed a loss of nearly 8 years in men and 4 years in women.

**Figure 2:**
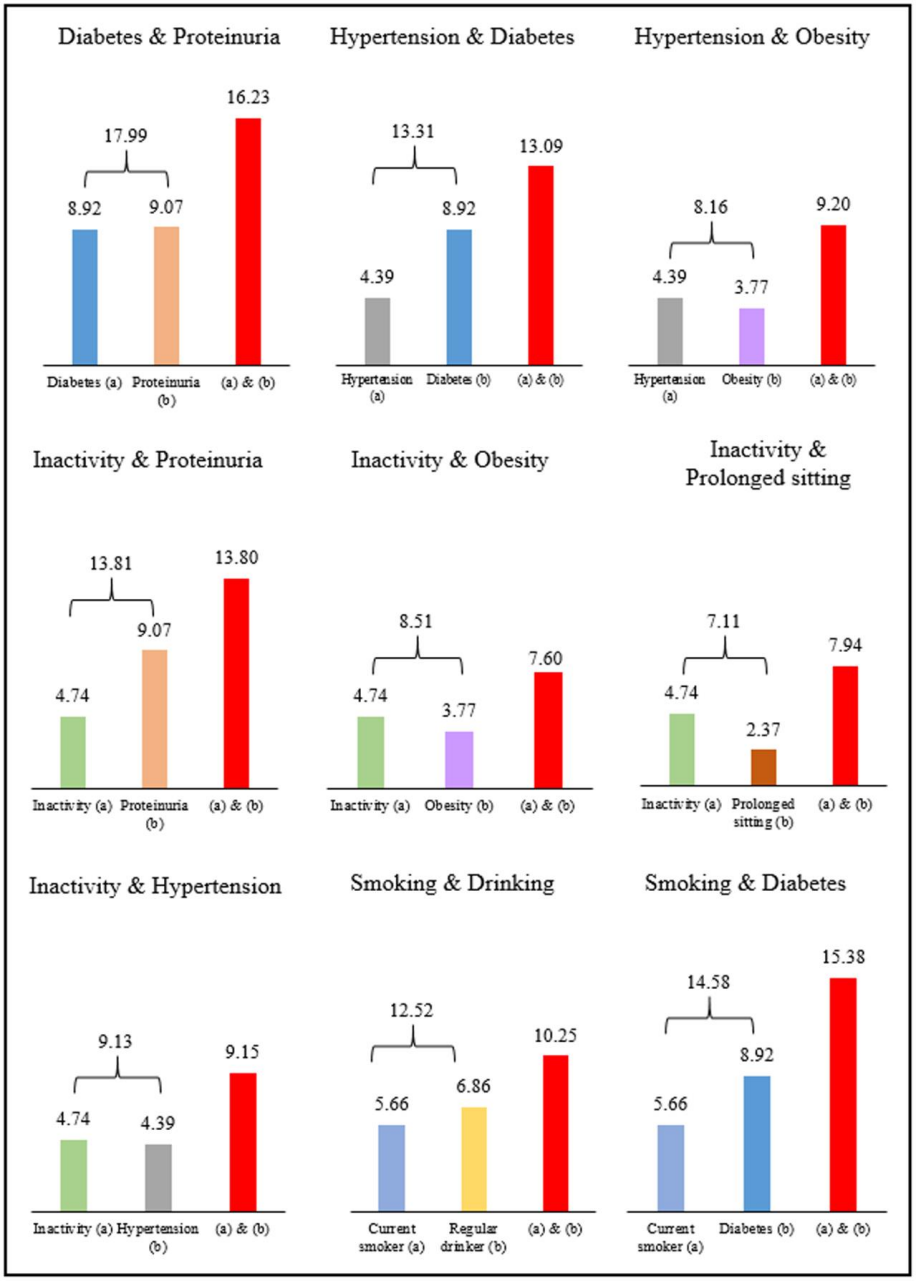
Years of life lost from two co-existing risk factors in men.

The loss of life expectancy for individuals with two co-existing risks is shown in Figure 2 for selected risks. Those having diabetes (8.9 years) and proteinuria (9.1 years) present at the same time showed a loss of 16.2 years, a number close to the sum of each risk. Similarly, inactive individuals (4.7 years) with hypertension (4.4 years) had a loss of 9.2 years, or with proteinuria (9.1 years) had a loss of 13.8 years.

## DISCUSSION

We identified 30 risks from data collected in routine medical screening. By converting these health risks into life expectancy loss, patients can then easily understand the magnitude of each of their risks. If interpreted by clinicians, risk communication can be greatly facilitated. Given the reality of multiple risks in life, one is usually at a loss as to their relative importance. For example, three commonly encountered risks in men: hypertension, diabetes, and high cholesterol, are often interpreted as having a 48%, 102% and 13% increase in all-cause mortality based on their hazard ratios of 1.48, 2.02 and 1.13, respectively. In contrast, the life expectancy loss of 4.4 years, 8.9 years, and 0.9 years, respectively, can make the implications obvious. Expressing the risk as years of lost life expectancy serves several purposes: simplifying the size of the risks, setting priorities for proactive action among competing interests, and motivating behavioral changes. The benefits of risk reduction will be in the number of years gained, which is an understandable term. It is a potential paradigm shift in risk communication that can facilitate preventive action during teachable moments.

The seemly additive feature of life years lost was intriguing, as these results were not expected [4]. Whether additive or not, in theory, should depend on the interdependencies or interactions between the two risks. Positive synergistic effects of certain coexisting risks, e.g., smoking and diabetes, have a greater impact on life expectancy loss than the sum of the individual conditions. On the other hand, overlapping risks, e.g., smoking and drinking have a smaller joint effect than the sum of the individual risks. Since the exact relationship between risks is not fully understood, their joint impact on life expectancy requires further studies. This finding highlights the seriousness of the combined impact of multiple risks and underscores the utility of life years lost, with some risks more life-threatening than others.

For the top 20 risks, an overwhelming majority of the study subjects, 9 out of 10, had at least one risk, losing 4-10 years of life, and over two-thirds had two or more, losing 8-16 years, and one-third had three risks, losing 12-25 years. At least in theory, two-thirds of the cohort could gain up to 16 years if their coexisting two risks were substantially modified. Armed with this information, health educators or clinicians can better motivate patients to set priorities and make behavioral changes.

The magnitude of life expectancy loss observed in this study was generally consistent with the literature. For example, among the top 10 risks, the loss from diabetes, at 9 years, proteinuria, at 9-10 years, or COPD, at 6 years, are in line with those reported [11–18]. Much of the literature on life expectancy loss was limited to cardiovascular risk factors [5, 7, 8, 11–13, 16–18]. With regard to some non-traditional risks like high heart rate, elevated CRP, or high white count, only increased hazard ratios [19–22], not losses of life expectancy, were available.

While each of the 30 health risks is important, we focused on the top ten risk factors for males and females. In addition, a few particular risks that may have general interests are also selected for discussion.

### High heart rate

The independent relationship between resting heart rate and mortality has been reported [23, 24]. Heart rate reflects the known relationship between cardiorespiratory fitness and mortality since low heart rates are characteristic of fit individuals. For those with a normal heart rate on the high side, 80-99 beats/min, a large loss of 5-8 years was found. Rapid heartbeat overloads the heart, with an extra 300 million beats in a span of 20 years for those with 90 beats/min compared to those with 60 beats/min. Indeed, heart rate has been inversely associated with longevity across mammal species, from rats, with 500 beats/min for 2 years, to whales, with 10 beats/min for 80 years. [23, 24].

### Proteinuria and low GFR

Proteinuria and low GFR were components of chronic kidney disease. With proteinuria prevalence at 7-8%, its large life-shortening effect of 9-10 years has often been overlooked [25, 26].^.^ Most with proteinuria are unaware of such a risk, a risk easily detected by urine dipstick [26, 27]. The only report on the risk of proteinuria in the form of life-shortening came from Canadian insurance data, with results similar to our study [28]. The prevalence of low GFR increased with age. The life shortening is about 4 to 7 years. Low eGFR and proteinuria, when found in diabetics, is known as diabetic kidney disease (DKD). DKD has been reported to shorten life expectancy by up to 16 years, as the presence of proteinuria made diabetes behave like a different disease [29].

### Diabetes

The 9 years of life lost from diabetes is much higher than hypertension or elevated cholesterol. The differences in life loss observed between diabetic and nondiabetic participants were similar to those found in previous studies [30–32]. In the Framingham Heart Study, diabetic men and women 50 years and older lived on average 7.5 and 8.2 years less than their nondiabetic equivalents [31].

### Inflammatory markers: CRP and WBC

Both CRP and WBC count are well-known inflammatory markers, with increased mortality for all causes and for CVD [19–22]. In clinical settings, each of these two risks are routinely measured, but the large loss of life expectancy, 5-7 years for CRP (≥ 3.0 mg/L) and 6-7 years for WBC (≥ 9000 / mm^3^) was surprising and has not previously been reported.

### Anemia

The prevalence of mild anemia (hemoglobin 10 - 13.4 g/dL) is 5.3% for males and 10.2% for females. Anemia was primarily caused by iron deficiency; low oxygen-carrying capacity may result from other chronic cardiovascular diseases. Though the prevalence for females is higher than for males, the loss of life expectancy for males has higher impact at 7.5 years than for females at 4.9 years.

### Obesity or underweight

The 3-4 years of life lost from obese individuals in this cohort is slightly smaller than those reported for Western populations [33]. In contrast, men who are underweight had a loss of 7 years, much larger than obese men. Such a paradoxical observation with underweight worse than obesity has been reported from Japan [34]. Underweight, quite common in Asian women (12%), had a loss of 4.3 years, larger than their obese counterparts of 2.6 years.

### Smoking, regular drinking, and betel quid chewing

Smoking, drinking, and betel quid chewing were three major lifestyle risk factors for males. These were all high-risk factors for several cancer sites. The life loss of a regular drinker is slightly larger than betel quid chewing or smokers and may be due to accidents from drunk driving. Most betel quid chewers were also smokers and they got similar loss of life of 5 to 6 years.

### COPD

COPD is not only a risk factor for lung diseases but also a systemic factor for several causes of death [35, 36]. COPD has 6 years of life loss but when concurrent with smoking, the years of life lost may increase to ten or more years.

### Hypertension

In a recent publication of Global Burden of Disease Study (2020), high systolic blood pressure (SBP) was, among 87 risk factors analyzed, the leading risk factor for attributable deaths globally [37]. A similar conclusion a decade ago was that high BP was the biggest single contributor to all-cause mortality [38, 39]. However, the life-shortening effect ranked it about the tenth risk factor in this cohort with 5 to 6 years of life loss.

### Sleep duration

Longer than 8 hours of sleep duration has been reported as an independent risk for mortality [40, 41]. It was equivalent to a 5-year loss of life in this study. The mechanism, however, is not well known but may reflect some underlying health conditions. Hazard ratios of sleep shorter than 4 hours were comparable to those of longer than 8 hours (Supplementary Table 1), suggesting that 6 to 7 hours of sleep a day is the goal to achieve.

### Low and high total cholesterol

The minimal loss of life years (1 year) for high cholesterol (≥ 240 mg/dL) and a larger loss (4-5 years) for low cholesterol (<160 mg/dL) may seem puzzling but has been repeatedly reported among Asians [42, 43]. High cholesterol is known to be a major risk for heart disease. However, the relative proportion of heart disease among Asians was small, with heart disease mortality in Taiwan (11.7%) half that in the United States (23.1%). Low cholesterol was known to have increased mortality from hemorrhagic stroke and from liver cancer, which has been reported in other Asian populations [42, 43].

### Prolonged sitting

Prolonged sitting has received increasing attention and was reported as a mortality risk independent of physical activity. [44, 45] Prolonged sitting could shorten one’s life by nearly 8 years when coupled with physical inactivity. Prolonged sitting and inactivity were the two common health risks, and their personal and public health implication cannot be overemphasized.

There are some limitations to this study. First, the loss of life expectancy was calculated without adjusting for confounding factors. However, adjusting life expectancy for comorbidities is not commonly done and is more of an academic exercise. Second, the life-shortening effect was calculated from differences in life expectancy, and technically speaking, different health risks based on different reference groups cannot be directly compared. Third, the life expectancy from this study is cohort-specific and may not be applicable to other populations, such as non-Asians. However, our study, by focusing on the life expectancy differences, should have minimized cohort-specific concerns [5]. Fourth, risk factors were determined from initial entry to the cohort and may have changed during the study. However, with such a large set of data, its impact would be very limited [25, 46].

In conclusion, behavioral modification to reduce health risks is of prime importance in daily clinical practice, and yet, effective counseling has been difficult. Life-year loss, when used to represent the size of each risk, would offer a different perspective and should be readily understandable to patients to prioritize treatment strategy. This new mode of practice can be a paradigm shift in conducting effective clinical management. For the top 20 risks, 9 out of 10 individuals had at least one risk, losing 4-10 years, and over two-thirds had two, losing 8-16 years, and one-third had three risks, losing 12-25 years. The message of loss of life expectancy is more intuitive and could be a powerful motivator for behavior changes.

## MATERIALS AND METHODS

### Study population

The study cohort consisted of 543,410 adults, age 20 or older, who participated in a comprehensive health screening program run by a private firm, MJ Health Management Institution, Taipei, Taiwan. MJ attracted self-paying participants from all over Taiwan. All participants paid to become members and to have health examinations and may appear to be of higher socioeconomic status than the average population. However, many members paid for their parents and relatives, who could have been in different or lower socioeconomic classes. MJ also accepted individuals paid for by different companies, constituting occupational cohorts. A detailed description of this cohort has been reported elsewhere [47]. This cohort is an open (dynamic) cohort, and study subjects have been successively recruited from all walks of life since 1994. Every individual’s identification number was matched with the National Death file between 1994 and 2008.

A self-administered questionnaire gathered demographic, lifestyle, and medical history information, including levels of physical activity, developed from frequency, duration, and intensity information [25, 46]. Physical activity in this study referred to leisure-time physical activity only [46].

All clinics in the program used a centralized laboratory for consistency. Overnight fasting blood was collected and analyzed by a Hitachi 7150 auto-analyzer for a standard panel of blood tests. EKG recording for heart rate, dipstick for proteinuria, and spirometry for lung function were carried out, with details published elsewhere [25, 46].

### Health risks

Selected health risks included behavioral risks and cardio-metabolic medically screened risks. The definitions, cut-points, and reference group for each risk are listed in Table 3. Selection of the risks for this study was based on: (1) finding a statistically significant hazard ratio (HR) for mortality; and (2) meeting a minimum requirement of a prevalence of 5% in either men or women in the cohort. The latter was set up to assure that the risk was commonly encountered, with at least one found in 20 individuals.

**Table 3:** Definition and cut-points of the 30 risk factors.

Risk factor*		**Risk group**		**Reference group**
High heart rate		≥ 90 beats/minute		60 - 69 beats/minute
Proteinuria		Trace or positive (≥ 1+) (by dipstick)		Negative
Diabetes		Fasting blood glucose ≥ 126 mg/dL or on medication		Fasting blood glucose 90 - 109 mg/dL
Elevated white blood cell (WBC)		≥ 9,000 / mm^3^		4000 - 5999/mm3
Mild anemia		Hemoglobin 10 - 13.4 g/dL in men; 10 - 11 g/dL in women		Hemoglobin 13.5 -15 g/dL in men; 12 - 14 g/dL in women
C-reactive protein		≥ 3.0 mg/L		< 1 mg/L
Underweight		Men: BMI < 19 kg/m^2^; Women: BMI < 18.5 kg/m^2^		BMI 23 - 24 kg/m^2^
Regular drinker		Alcohol drinking: ≥ 2 drinks, ≥ 3 times a week		Non-drinker
Chronic Obstructive Pulmonary Disease (COPD)		GOLD definition: FEV_1_/FVC < 0.7		FEV_1_/FVC ≥ 0.7 and FVC ≥ 80%
Betel quid chewing		Chewer		Never chewer
Current smoker		Current smoker		Never smoker
Restrictive lung disease		GOLD definition: FEV_1_/FVC ≥ 0.7, but FVC < 80%		FEV_1_/FVC ≥ 0.7 and FVC ≥ 80%
Long-duration sleep		Sleep duration > 8 hours/day		Sleep duration 6 - 7 hours/day
Physical inactivity		< 3.75 MET-h/wk (inactive)		≥ 7.5 MET-h/wk
Hepatitis B carrier (HBsAg)		Hepatitis B surface antigen positive (HBsAg)		Surface antigen negative
Hypertension (SBP)		Systolic blood pressure ≥ 140 mmHg or on hypertension medication		Systolic blood pressure 90 - 119 mmHg
Hypertension (DBP)		Diastolic blood pressure ≥ 90 mmHg or on hypertension medication		Diastolic blood pressure 60 - 79 mmHg
Low cholesterol		Total cholesterol < 160 mg/dL		Total cholesterol 180 - 199 mg/dL
Obesity		BMI ≥ 30 kg/m^2^		BMI 23 - 24 kg/m^2^
Low glomerular filtration rate		< 60 ml/min/1.73m^2^ (by CKD-EPI definition)		60 - 89 ml/min/1.73m^2^ (by CKD-EPI definition)
Elevated aspartate aminotransferase (AST)		≥ 25 U/L		< 25 U/L
Low blood glucose		Fasting blood glucose < 90 mg/dL		Fasting blood glucose 90 - 109 mg/dL
High uric acid		≥ 7 mg/dL		5.0 – 5.9 mg/dL
Low HDL-C		HDL-C < 35 mg/dL		HDL-C 35 - 69 mg/dL
High triglyceride		Triglyceride ≥ 200 mg/dL		Triglyceride < 100 mg/dL
Prolonged sitting		Yes (≥ 8 hours/day)		No (< 8 hours/day)
Metabolic Syndrome (ATP III definition)		When 3 or more of the following characteristics present: Waistline ≥ 90 cm for men, or ≥ 80 cm for women Triglyceride ≥ 150 mg/dL. HDL-C <40 mg/dL for male, or <50 mg/dL for female. Blood pressure ≥ 130 (SBP) / ≥ 85 (DBP) mm Hg. Fasting blood glucose ≥ 110 mg/dL.		No metabolic syndrome
Pre-diabetes		Fasting blood glucose 110 - 125 mg/dL		Fasting blood glucose 90 - 109 mg/dL
High cholesterol		Total cholesterol ≥ 240 mg/dL		Total cholesterol 180 - 199 mg/dL
Pre-hypertension		Systolic blood pressure 120 - 139 mmHg		Systolic blood pressure 90 - 119 mmHg

*Listed in the order according to Table 2.

### Statistical analysis

Life expectancy is one of the most widely used demographic measures, which summarize age-specific mortality rates into one number. The calculation of life expectancy and its confidence interval were carried out using the life table method developed by Chiang [48]. Confidence intervals for the year of life loss was calculated based on a comparison of two population means, i.e., life expectancies, using the standard independent t-test.

The life expectancy calculated in this study referred to the average remaining years a 30-year-old person would be expected to live. Those with two coexisting risks, for selected risk factors, were compared against those with neither risk. The above analysis was limited to men, due to low-risk prevalence and smaller numbers of deaths in women.

The hazard ratio for each risk factor for men and for women was also calculated using Cox regression for both univariate (adjusted for age only) and multivariate (adjusted for age, smoking, BMI, systolic blood pressure, fasting blood glucose, and total cholesterol) analysis. All statistical analyses were performed with SAS, version 9.4 (SAS Institute, Cary, NC, USA).

## Supplementary Material

Supplementary Table 1

Supplementary Table 2
