# Supplementary material for: Converting health risks into loss of life years - a paradigm shift in clinical risk communication
Source: Aging (Albany NY). 2021 Sep 7;13(17):21513–25. doi: 10.18632/aging.203491 (PMC8457574; doi:10.18632/aging.203491)
Supplement: Supplementary Table 1 [file aging-13-203491-s001.docx]

Supplementary Table 1. Age-adjusted and multi-variable adjusted hazard ratios for all-cause mortality by gender.

|  |  | **Men** | | | | | |  | **Women** | | | | | |
| --- | --- | --- | --- | --- | --- | --- | --- | --- | --- | --- | --- | --- | --- | --- |
| **Risk factor** | | (%) | Deaths | HR_1_ | (95% CI) | HR_2_ | (95% CI) |  | (%) | Deaths | HR_1_ | (95% CI) | HR_2_ | (95% CI) |
|  |  | 258,783 | 11,292 |  | |  | |  | 284,627 | 7,455 |  | |  | |
| **Resting heart rate** | |  |  |  |  |  |  |  |  |  |  |  |  |  |
|  | 40 - 59 beats/minute | 10.9% | 8.4% | 1.01 | (0.93-1.08) | 0.98 | (0.89-1.07) |  | 5.5% | 3.9% | 1.04 | (0.92-1.18) | 0.98 | (0.83-1.15) |
|  | 60 - 69 beats/minute | 32.6% | 25.5% | 1.00 | (reference) | 1.00 | (reference) |  | 27.0% | 19.6% | 1.00 | (reference) | 1.00 | (reference) |
|  | 70 - 79 beats/minute | 34.2% | 31.9% | 1.09 | (1.03-1.14)* | 1.07 | (1.01-1.14)* |  | 39.3% | 35.0% | 1.10 | (1.02-1.17)* | 1.10 | (1.01-1.20)* |
|  | 80 - 89 beats/minute | 16.1% | 21.0% | 1.38 | (1.31-1.46)* | 1.32 | (1.23-1.42)* |  | 20.4% | 24.8% | 1.34 | (1.25-1.44)* | 1.25 | (1.14-1.37)* |
|  | ≥ 90 beats/minute | 6.1% | 13.3% | 2.16 | (2.03-2.30)* | 2.10 | (1.94-2.27)* |  | 7.9% | 16.7% | 2.00 | (1.85-2.16)* | 1.66 | (1.49-1.84)* |
|  | 90 - 99 beats/minute | 4.6% | 8.8% | 1.94 | (1.80-2.08)* | 1.90 | (1.73-2.08)* |  | 5.9% | 10.8% | 1.81 | (1.66-1.97)* | 1.48 | (1.32-1.67)* |
|  | ≥ 100 beats/minute | 1.5% | 4.5% | 2.78 | (2.53-3.06)* | 2.67 | (2.36-3.01)* |  | 2.0% | 5.9% | 2.53 | (2.27-2.81)* | 2.09 | (1.81-2.43)* |
| **Proteinuria** | |  |  |  |  |  |  |  |  |  |  |  |  |  |
|  | Negative (-) | 92.1% | 77.7% | 1.00 | (reference) | 1.00 | (reference) |  | 92.9% | 77.5% | 1.00 | (reference) | 1.00 | (reference) |
|  | Trace (±) or Positive ( ≥ 1+) | 7.9% | 22.3% | 2.11 | (2.02-2.20)* | 2.01 | (1.90-2.13)* |  | 7.1% | 22.5% | 2.44 | (2.31-2.58)* | 2.18 | (2.02-2.35)* |
|  | Trace (±) | 5.9% | 11.5% | 1.72 | (1.62-1.82)* | 1.69 | (1.57-1.81)* |  | 5.3% | 9.9% | 1.80 | (1.67-1.95)* | 1.73 | (1.57-1.91)* |
|  | Positive (1+) | 1.3% | 5.5% | 2.23 | (2.05-2.42)* | 2.33 | (2.07-2.63)* |  | 1.1% | 6.4% | 2.72 | (2.47-2.99)* | 2.34 | (2.02-2.71)* |
|  | Positive ( ≥ 2+) | 0.8% | 5.4% | 3.74 | (3.44-4.07)* | 3.89 | (3.47-4.36)* |  | 0.7% | 6.2% | 4.61 | (4.18-5.08)* | 4.91 | (4.26-5.65)* |
| **Fasting blood glucose** | |  |  |  |  |  |  |  |  |  |  |  |  |  |
|  | < 90 mg/dL | 17.5% | 17.2% | 1.31 | (1.24-1.38)* | 1.27 | (1.18-1.36)* |  | 30.8% | 16.8% | 1.13 | (1.06-1.20)* | 1.06 | (0.97-1.17) |
|  | 90 - 109 mg/dL | 69.9% | 54.4% | 1.00 | (reference) | 1.00 | (reference) |  | 59.9% | 50.9% | 1.00 | (reference) | 1.00 | (reference) |
|  | 110 - 125 mg/dL | 6.9% | 9.7% | 1.15 | (1.08-1.22)* | 1.20 | (1.11-1.30)* |  | 4.5% | 8.9% | 1.13 | (1.04-1.22)* | 1.02 | (0.91-1.15) |
|  | ≥ 126 mg/dL or on medicine | 5.7% | 18.7% | 2.02 | (1.92-2.13)* | 2.02 | (1.89-2.15)* |  | 4.8% | 23.4% | 2.40 | (2.26-2.54)* | 2.24 | (2.07-2.42)* |
| **White blood cell** | |  |  |  |  |  |  |  |  |  |  |  |  |  |
|  | < 4000 / mm^3^ | 2.1% | 2.8% | 1.51 | (1.35-1.70)* | 1.53 | (1.31-1.78)* |  | 5.1% | 5.3% | 1.46 | (1.31-1.62)* | 1.57 | (1.37-1.79)* |
|  | 4000 - 5999 / mm^3^ | 36.3% | 28.1% | 1.00 | (reference) | 1.00 | (reference) |  | 45.2% | 35.7% | 1.00 | (reference) | 1.00 | (reference) |
|  | 6000 - 8999 / mm^3^ | 52.7% | 54.1% | 1.23 | (1.18-1.29)* | 1.11 | (1.05-1.17)* |  | 44.1% | 47.2% | 1.15 | (1.10-1.21)* | 1.08 | (1.01-1.16)* |
|  | ≥ 9000 / mm^3^ | 9.0% | 15.0% | 1.82 | (1.71-1.93)* | 1.57 | (1.45-1.69)* |  | 5.7% | 11.8% | 1.90 | (1.76-2.05)* | 1.68 | (1.52-1.87)* |
| **Hemoglobin** | |  |  |  |  |  |  |  |  |  |  |  |  |  |
|  | Low (<10) | 0.2% | 1.5% | 3.60 | (3.09-4.19)* | 3.71 | (3.04-4.54)* |  | 1.6% | 3.6% | 3.13 | (2.77-3.54)* | 3.40 | (2.89-4.02)* |
|  | Mild (Men 10-<13.5; Women 10-<12) | 5.3% | 18.1% | 1.56 | (1.49-1.64)* | 1.70 | (1.59-1.82)* |  | 10.2% | 15.5% | 1.52 | (1.43-1.62)* | 1.67 | (1.53-1.82)* |
|  | Normal | 66.1% | 64.0% | 1.00 | (reference) | 1.00 | (reference) |  | 85.1% | 76.0% | 1.00 | (reference) | 1.00 | (reference) |
|  | High (Men ≥16; Women ≥15) | 28.4% | 16.4% | 1.03 | (0.98-1.08) | 0.93 | (0.87-1.00)* |  | 3.0% | 5.0% | 1.38 | (1.25-1.54)* | 1.21 | (1.04-1.40)* |

HR_1_: Age-adjusted hazard ratio.

HR_2_: Multi-variable adjusted hazard ratio.

Multi-variable adjusted hazard ratios are adjusted for age, smoking, body mass index, systolic blood pressure, fasting blood glucose, and total cholesterol in a multivariate Cox model when appropriate.

|  |  | **Men** | | | | | | |  | **Women** | | | | | |
| --- | --- | --- | --- | --- | --- | --- | --- | --- | --- | --- | --- | --- | --- | --- | --- |
| **Risk factor** | | N(%) | | Deaths | HR_1_ | (95% CI) | HR_2_ | (95% CI) |  | N(%) | Deaths | HR_1_ | (95% CI) | HR_2_ | (95% CI) |
|  |  | 258,783 | | 11,292 |  | |  | |  | 284,627 | 7,455 |  | |  | |
| **C-reactive protein** | | |  |  |  |  |  |  |  |  |  |  |  |  |  |
|  | < 1.0 mg/L | | 70.4% | 52.1% | 1.00 | (reference) | 1.00 | (reference) |  | 72.3% | 52.1% | 1.00 | (reference) | 1.00 | (reference) |
|  | 1.0 - 2.9 mg/L | | 18.5% | 21.9% | 1.21 | (1.14-1.28)* | 1.24 | (1.17-1.32)* |  | 17.2% | 22.1% | 1.12 | (1.04-1.20)* | 1.20 | (1.11-1.29)* |
|  | ≥ 3.0 mg/L | | 11.1% | 26.0% | 1.84 | (1.75-1.94)* | 1.83 | (1.72-1.94)* |  | 10.5% | 25.8% | 1.59 | (1.49-1.70)* | 1.59 | (1.47-1.72)* |
|  | 3.0 - 4.9 mg/L | | 5.0% | 8.3% | 1.48 | (1.36-1.60)* | 1.49 | (1.37-1.62)* |  | 4.9% | 9.7% | 1.38 | (1.26-1.52)* | 1.39 | (1.25-1.55)* |
|  | 5.0 - 9.9 mg/L | | 3.1% | 7.8% | 1.94 | (1.79-2.11)* | 1.95 | (1.78-2.13)* |  | 3.1% | 7.5% | 1.50 | (1.35-1.66)* | 1.51 | (1.34-1.70)* |
|  | ≥ 10 mg/L | | 3.0% | 10.0% | 2.20 | (2.05-2.38)* | 2.15 | (1.98-2.33)* |  | 2.6% | 8.7% | 2.04 | (1.85-2.25)* | 2.03 | (1.81-2.27)* |
| **Body mass index** | |  | |  |  |  |  |  |  |  |  |  |  |  |  |
|  | < 18.5 kg/m^2^ (women) |  | |  |  |  |  |  |  | 12.0% | 5.3% | 1.46 | (1.30-1.63)* | 1.64 | (1.42-1.89)* |
|  | < 19 kg/m^2^ (men) | 6.3% | | 9.0% | 1.57 | (1.46-1.69)* | 1.88 | (1.71-2.07)* |  |  |  |  |  |  |  |
|  | 18.5 - 22 kg/m^2^ (women) |  | |  |  |  |  |  |  | 50.6% | 33.2% | 1.06 | (0.99-1.13) | 1.14 | (1.04-1.24)* |
|  | 19 - 22 kg/m^2^ (men) | 31.3% | | 30.3% | 1.13 | (1.08-1.19)* | 1.17 | (1.10-1.25)* |  |  |  |  |  |  |  |
|  | 23 - 24 kg/m^2^ | 26.3% | | 24.7% | 1.00 | (reference) | 1.00 | (reference) |  | 16.1% | 21.1% | 1.00 | (reference) | 1.00 | (reference) |
|  | 25 - 29 kg/m^2^ | 31.5% | | 31.6% | 1.05 | (0.99-1.10) | 0.98 | (0.91-1.04) |  | 17.7% | 32.3% | 1.09 | (1.02-1.16)* | 0.99 | (0.91-1.08) |
|  | ≥ 30 kg/m^2^ | 4.5% | | 4.4% | 1.53 | (1.39-1.69)* | 1.26 | (1.11-1.42)* |  | 3.6% | 8.1% | 1.41 | (1.28-1.55)* | 1.23 | (1.08-1.40)* |
| **Alcohol drinking** | |  | |  |  |  |  |  |  |  |  |  |  |  |  |
|  | Non drinker | 64.8% | | 49.8% | 1.00 | (reference) | 1.00 | (reference) |  | 90.9% | 90.3% | 1.00 | (reference) | 1.00 | (reference) |
|  | Moderate drinker | 23.2% | | 24.4% | 0.96 | (0.91-1.02) | 0.87 | (0.82-0.93)* |  | 6.7% | 6.3% | 0.88 | (0.78-1.00) | 0.88 | (0.77-1.02) |
|  | Regular drinker | 12.1% | | 25.8% | 1.79 | (1.69-1.90)* | 1.52 | (1.43-1.61)* |  | 2.4% | 3.4% | 1.77 | (1.50-2.10)* | 1.45 | (1.21-1.73)* |
| **Lung function** | |  | |  |  |  |  |  |  |  |  |  |  |  |  |
|  | Normal | 78.1% | | 46.4% | 1.00 | (reference) | 1.00 | (reference) |  | 75.7% | 45.5% | 1.00 | (reference) | 1.00 | (reference) |
|  | Restrictive lung disease | 14.6% | | 32.3% | 1.55 | (1.48-1.62)* | 1.52 | (1.44-1.60)* |  | 18.1% | 38.4% | 1.45 | (1.38-1.53)* | 1.43 | (1.34-1.53)* |
|  | COPD | 7.3% | | 21.3% | 1.59 | (1.51-1.67)* | 1.56 | (1.46-1.68)* |  | 6.3% | 16.1% | 1.45 | (1.35-1.55)* | 1.42 | (1.28-1.58)* |
| **Betel quid chewing** | |  | |  |  |  |  |  |  |  |  |  |  |  |  |
|  | Never chewer | 81.2% | | 78.1% | 1.00 | (reference) | 1.00 | (reference) |  | 99.3% | 97.8% | 1.00 | (reference) | 1.00 | (reference) |
|  | Chewer | 18.8% | | 21.9% | 1.82 | (1.72-1.93)* | 1.56 | (1.46-1.66)* |  | 0.7% | 2.2% | 2.09 | (1.70-2.56)* | 1.54 | (1.22-1.94)* |
| **Smoking** | |  | |  |  |  |  |  |  |  |  |  |  |  |  |
|  | Never smoker | 48.6% | | 31.3% | 1.00 | (reference) | 1.00 | (reference) |  | 91.6% | 89.3% | 1.00 | (reference) | 1.00 | (reference) |
|  | Ex-smoker | 10.9% | | 20.5% | 1.36 | (1.27-1.45)* | 1.36 | (1.27-1.45)* |  | 1.6% | 2.8% | 1.70 | (1.41-2.04)* | 1.74 | (1.44-2.09)* |
|  | Current smoker | 40.6% | | 48.2% | 1.69 | (1.60-1.79)* | 1.76 | (1.67-1.86)* |  | 6.7% | 7.9% | 1.79 | (1.59-2.00)* | 1.87 | (1.67-2.09)* |
| **Sleep duration** | |  | |  |  |  |  |  |  |  |  |  |  |  |  |
|  | < 4 hours/day | 0.9% | | 2.4% | 1.49 | (1.28-1.73)* | 1.49 | (1.28-1.74)* |  | 1.3% | 4.1% | 1.35 | (1.16-1.57)* | 1.32 | (1.12-1.55)* |
|  | 4-5 hours/day | 19.3% | | 21.0% | 1.06 | (1.00-1.13)* | 1.07 | (1.01-1.14)* |  | 19.7% | 26.5% | 1.03 | (0.96-1.10) | 1.03 | (0.95-1.11) |
|  | 6-7 hours/day | 71.0% | | 56.7% | 1.00 | (reference) | 1.00 | (reference) |  | 68.1% | 53.5% | 1.00 | (reference) | 1.00 | (reference) |
|  | > 8 hours/day | 8.9% | | 19.9% | 1.43 | (1.34-1.52)* | 1.36 | (1.28-1.45)* |  | 10.9% | 15.9% | 1.44 | (1.33-1.57)* | 1.37 | (1.26-1.50)* |

HR_1_: Age-adjusted hazard ratio.

HR_2_: Multi-variable adjusted hazard ratio.

Multi-variable adjusted hazard ratios are adjusted for age, smoking, body mass index, systolic blood pressure, fasting blood glucose, and total cholesterol in a multivariate Cox model when appropriate.

|  |  | | **Men** | | | | | | | | | | | |  | | **Women** | | | | | | | | | |
| --- | --- | --- | --- | --- | --- | --- | --- | --- | --- | --- | --- | --- | --- | --- | --- | --- | --- | --- | --- | --- | --- | --- | --- | --- | --- | --- |
| **Risk factor** | | | N(%) | | Deaths | | HR_1_ | | (95% CI) | | HR_2_ | | (95% CI) | |  | | N(%) | | Deaths | | HR_1_ | | (95% CI) | | HR_2_ | (95% CI) |
|  |  | | 258,783 | | 11,292 | |  | | | |  | | | |  | | 284,627 | | 7,455 | |  | | | |  | |
| **Physical activity** | |  | |  | |  | |  | |  | |  | |  | |  | |  | |  | |  | |  | |  |
|  | <3.75 MET-h/wk (Inactive) | 48.4% | | 51.6% | | 1.55 | | (1.47-1.63)* | | 1.47 | | (1.39-1.55)* | |  | | 59.9% | | 54.2% | | 1.36 | | (1.27-1.45)* | | 1.31 | | (1.21-1.40)* |
|  | 3.75 - 7.49 MET-h/wk | 22.4% | | 16.3% | | 1.19 | | (1.11-1.27)* | | 1.17 | | (1.09-1.25)* | |  | | 21.4% | | 19.2% | | 1.19 | | (1.09-1.29)* | | 1.19 | | (1.09-1.30)* |
|  | ≥ 7.5 MET-h/wk | 29.2% | | 32.1% | | 1.00 | | (reference) | | 1.00 | | (reference) | |  | | 18.7% | | 26.7% | | 1.00 | | (reference) | | 1.00 | | (reference) |
| **Hepatitis B surface antigen** | | |  | |  | |  | |  | |  | |  | |  | |  | |  | |  | |  | |  |  |
|  | Negative (-) | | 82.6% | | 82.5% | | 1.00 | | (reference) | | 1.00 | | (reference) | |  | | 87.6% | | 87.0% | | 1.00 | | (reference) | | 1.00 | (reference) |
|  | Positive (+) | | 17.4% | | 17.5% | | 1.55 | | (1.47-1.63)* | | 1.62 | | (1.52-1.72)* | |  | | 12.4% | | 13.0% | | 1.35 | | (1.26-1.44)* | | 1.36 | (1.24-1.49)* |
| **Systolic blood pressure** | | |  | |  | |  | |  | |  | |  | |  | |  | |  | |  | |  | |  |  |
|  | < 90 mmHg | | 0.5% | | 0.5% | | 1.17 | | (0.89-1.54) | | 1.06 | | (0.72-1.56) | |  | | 2.3% | | 0.7% | | 1.16 | | (0.88-1.52) | | 1.07 | (0.73-1.56) |
|  | 90 - 119 mmHg | | 43.8% | | 23.3% | | 1.00 | | (reference) | | 1.00 | | (reference) | |  | | 59.6% | | 21.5% | | 1.00 | | (reference) | | 1.00 | (reference) |
|  | 120 - 139 mmHg | | 36.7% | | 28.3% | | 1.12 | | (1.06-1.18)* | | 1.12 | | (1.05-1.20)* | |  | | 21.7% | | 22.6% | | 1.05 | | (0.98-1.13) | | 0.98 | (0.88-1.08) |
|  | ≥ 140 mmHg or on medicine | | 19.1% | | 48.0% | | 1.48 | | (1.41-1.56)* | | 1.54 | | (1.45-1.65)* | |  | | 16.4% | | 55.1% | | 1.45 | | (1.36-1.55)* | | 1.36 | (1.24-1.49)* |
| **Diastolic blood pressure** | | |  | |  | |  | |  | |  | |  | |  | |  | |  | |  | |  | |  |  |
|  | < 60 mmHg | | 6.0% | | 4.2% | | 1.15 | | (1.05-1.27)* | | 1.11 | | (0.98-1.26) | |  | | 17.2% | | 5.1% | | 1.15 | | (1.04-1.29)* | | 1.12 | (0.97-1.29) |
|  | 60 - 79 mmHg | | 58.0% | | 35.0% | | 1.00 | | (reference) | | 1.00 | | (reference) | |  | | 57.4% | | 30.1% | | 1.00 | | (reference) | | 1.00 | (reference) |
|  | 80 - 89 mmHg | | 14.0% | | 10.1% | | 1.05 | | (0.99-1.13) | | 1.00 | | (0.92-1.10) | |  | | 7.8% | | 7.3% | | 1.10 | | (1.00-1.21)* | | 1.15 | (1.01-1.31)* |
|  | ≥ 90 mmHg or on medicine | | 22.0% | | 50.8% | | 1.40 | | (1.34-1.46)* | | 1.45 | | (1.37-1.53)* | |  | | 17.6% | | 57.4% | | 1.48 | | (1.40-1.57)* | | 1.44 | (1.34-1.56)* |
| **Total cholesterol** | | |  | |  | |  | |  | |  | |  | |  | |  | |  | |  | |  | |  |  |
|  | < 160 mg/dL | | 17.0% | | 18.2% | | 1.39 | | (1.31-1.47)* | | 1.45 | | (1.34-1.57)* | |  | | 20.0% | | 14.9% | | 1.60 | | (1.48-1.74)* | | 1.85 | (1.65-2.07)* |
|  | 160 - 179 mg/dL | | 19.9% | | 18.0% | | 1.06 | | (1.00-1.13)* | | 1.05 | | (0.97-1.13) | |  | | 21.7% | | 14.5% | | 1.08 | | (1.00-1.17) | | 1.24 | (1.11-1.38)* |
|  | 180 - 199 mg/dL | | 22.6% | | 21.0% | | 1.00 | | (reference) | | 1.00 | | (reference) | |  | | 21.9% | | 18.6% | | 1.00 | | (reference) | | 1.00 | (reference) |
|  | 200 - 219 mg/dL | | 18.3% | | 17.2% | | 0.95 | | (0.90-1.01) | | 0.91 | | (0.84-0.98)* | |  | | 16.5% | | 17.5% | | 0.94 | | (0.87-1.01) | | 0.92 | (0.83-1.02) |
|  | 220 - 239 mg/dL | | 11.6% | | 12.0% | | 0.98 | | (0.92-1.05) | | 0.90 | | (0.83-0.98)* | |  | | 10.1% | | 13.8% | | 0.93 | | (0.86-1.01) | | 0.91 | (0.81-1.01) |
|  | ≥ 240 mg/dL | | 10.9% | | 13.9% | | 1.13 | | (1.06-1.20)* | | 1.08 | | (1.00-1.16) | |  | | 10.2% | | 20.9% | | 1.10 | | (1.02-1.18)* | | 0.99 | (0.90-1.09) |
| **Glomerular filtration rate** | | |  | |  | |  | |  | |  | |  | |  | |  | |  | |  | |  | |  |  |
|  | < 60 ml/min/1.73m^2^ | | 6.1% | | 30.3% | | 1.36 | | (1.30-1.43)* | | 1.39 | | (1.31-1.47)* | |  | | 4.3% | | 29.6% | | 1.78 | | (1.68-1.89)* | | 1.84 | (1.70-1.98)* |
|  | 60 - 89 ml/min/1.73m^2^ | | 55.4% | | 55.6% | | 1.00 | | (reference) | | 1.00 | | (reference) | |  | | 41.7% | | 50.9% | | 1.00 | | (reference) | | 1.00 | (reference) |
|  | ≥ 90 ml/min/1.73m^2^ | | 38.6% | | 14.1% | | 1.23 | | (1.16-1.30)* | | 1.19 | | (1.10-1.29)* | |  | | 54.0% | | 19.6% | | 0.96 | | (0.90-1.03) | | 1.00 | (0.91-1.09) |
| **Aspartate aminotransferase** | | |  | |  | |  | |  | |  | |  | |  | |  | |  | |  | |  | |  |  |
|  | < 25 U/L | | 64.1% | | 54.1% | | 1.00 | | (reference) | | 1.00 | | (reference) | |  | | 82.2% | | 62.0% | | 1.00 | | (reference) | | 1.00 | (reference) |
|  | ≥ 25 U/L | | 35.9% | | 45.9% | | 1.39 | | (1.34-1.45)* | | 1.41 | | (1.34-1.48)* | |  | | 17.8% | | 38.0% | | 1.48 | | (1.41-1.55)* | | 1.45 | (1.36-1.55)* |
|  | 25 - 39 U/L | | 28.2% | | 28.4% | | 1.08 | | (1.04-1.13)* | | 1.10 | | (1.04-1.16)* | |  | | 13.7% | | 22.3% | | 1.15 | | (1.09-1.22)* | | 1.16 | (1.08-1.25)* |
|  | ≥ 40 U/L | | 7.7% | | 17.5% | | 2.64 | | (2.51-2.78)* | | 2.65 | | (2.48-2.83)* | |  | | 4.1% | | 15.6% | | 2.51 | | (2.36-2.68)* | | 2.35 | (2.15-2.56)* |

HR_1_: Age-adjusted hazard ratio.

HR_2_: Multi-variable adjusted hazard ratio.

Multi-variable adjusted hazard ratios are adjusted for age, smoking, body mass index, systolic blood pressure, fasting blood glucose, and total cholesterol in a multivariate Cox model when appropriate.

|  |  | **Men** | | | | | |  | **Women** | | | | | |
| --- | --- | --- | --- | --- | --- | --- | --- | --- | --- | --- | --- | --- | --- | --- |
| **Risk factor** | | N(%) | Deaths | HR_1_ | (95% CI) | HR_2_ | (95% CI) |  | N(%) | Deaths | HR_1_ | (95% CI) | HR_2_ | (95% CI) |
|  |  | 258,783 | 11,292 |  | |  | |  | 284,627 | 7,455 |  | |  | |
| **Uric acid** | |  |  |  |  |  |  |  |  |  |  |  |  |  |
|  | < 5 mg/dL | 7.6% | 12.4% | 1.23 | (1.15-1.31)* | 1.25 | (1.15-1.36)* |  | 48.8% | 32.2% | 0.98 | (0.93-1.04) | 0.97 | (0.89-1.05) |
|  | 5 - 5.9 mg/dL | 19.3% | 19.4% | 1.00 | (reference) | 1.00 | (reference) |  | 28.8% | 26.3% | 1.00 | (reference) | 1.00 | (reference) |
|  | 6 - 6.9 mg/dL | 28.7% | 24.6% | 0.97 | (0.92-1.03) | 1.02 | (0.95-1.10)* |  | 13.9% | 19.4% | 1.12 | (1.04-1.19)* | 1.05 | (0.95-1.15) |
|  | ≥ 7 mg/dL | 44.4% | 43.6% | 1.10 | (1.05-1.16)* | 1.16 | (1.08-1.23)* |  | 8.5% | 22.0% | 1.39 | (1.30-1.49)* | 1.29 | (1.18-1.42)* |
|  | 7 – 7.9 mg/dL | 23.3% | 19.5% | 0.98 | (0.92-1.03) | 1.01 | (0.94-1.10) |  | 5.4% | 11.0% | 1.22 | (1.12-1.32)* | 1.12 | (1.00-1.25)* |
|  | ≥ 8 mg/dL | 21.1% | 24.0% | 1.23 | (1.16-1.30)* | 1.28 | (1.19-1.38)* |  | 3.1% | 11.0% | 1.62 | (1.50-1.76)* | 1.52 | (1.36-1.70)* |
| **HDL-C** | |  |  |  |  |  |  |  |  |  |  |  |  |  |
|  | < 35 mg/dL | 16.5% | 26.3% | 1.25 | (1.19-1.32)* | 1.25 | (1.18-1.32)* |  | 5.4% | 13.5% | 1.40 | (1.29-1.52)* | 1.29 | (1.18-1.42)* |
|  | 35 - 69 mg/dL | 78.5% | 67.4% | 1.00 | (reference) | 1.00 | (reference) |  | 75.4% | 86.5% | 1.00 | (reference) | 1.00 | (reference) |
|  | ≥ 70 mg/dL | 5.1% | 6.4% | 1.25 | (1.14-1.38)* | 1.21 | (1.10-1.34)* |  | 19.2% | 86.5% | 0.99 | (0.91-1.08) | 0.97 | (0.88-1.07) |
| **Triglyceride** | |  |  |  |  |  |  |  |  |  |  |  |  |  |
|  | < 100 mg/dL | 43.4% | 38.5% | 1.00 | (reference) | 1.00 | (reference) |  | 66.4% | 35.9% | 1.00 | (reference) | 1.00 | (reference) |
|  | 100 - 149 mg/dL | 27.3% | 28.3% | 0.98 | (0.4-1.03) | 0.94 | (0.89-1.00)* |  | 19.7% | 29.1% | 1.07 | (1.01-1.14)* | 0.98 | (0.90-1.06) |
|  | 150 - 199 mg/dL | 13.7% | 14.2% | 0.99 | (0.94-1.05) | 0.92 | (0.85-0.99)* |  | 7.5% | 15.9% | 1.14 | (1.06-1.22)* | 0.98 | (0.89-1.08) |
|  | ≥ 200 mg/dL | 15.7% | 19.0% | 1.21 | (1.15-1.28)* | 1.03 | (0.96-1.11) |  | 6.4% | 19.1% | 1.43 | (1.34-1.53)* | 1.15 | (1.04-1.26)* |
| **Prolonged sitting** | |  |  |  |  |  |  |  |  |  |  |  |  |  |
|  | Yes | 55.8% | 56.8% | 1.23 | (1.17-1.30)* | 1.20 | (1.14-1.27)* |  | 61.9% | 61.9% | 1.21 | (1.13-1.29)* | 1.15 | (1.07-1.23)* |
|  | No | 44.2% | 43.2% | 1.00 | (reference) | 1.00 | (reference) |  | 38.1% | 38.1% | 1.00 | (reference) | 1.00 | (reference) |
| **Metabolic Syndrome** | |  |  |  |  |  |  |  |  |  |  |  |  |  |
|  | No | 86.3% | 77.0% | 1.00 | (reference) | 1.00 | (reference) |  | 89.8% | 68.3% | 1.00 | (reference) | 1.00 | (reference) |
|  | Yes (ATP III) | 13.7% | 23.1% | 1.26 | (1.21-1.32)* | 1.46 | (1.38-1.54)* |  | 10.2% | 31.7% | 1.43 | (1.36-1.50)* | 1.55 | (1.44-1.66)* |
| **Hepatitis C antibody** | |  |  |  |  |  |  |  |  |  |  |  |  |  |
|  | Negative (-) | 97.6% | 86.0% | 1.00 | (reference) | 1.00 | (reference) |  | 97.1% | 78.5% | 1.00 | (reference) | 1.00 | (reference) |
|  | Positive (+) | 2.4% | 14.0% | 2.48 | (2.02-3.04)* | 2.44 | (1.96-3.04)* |  | 2.9% | 21.5% | 2.71 | (2.11-3.49)* | 2.14 | (1.59-2.88)* |

HR_1_: Age-adjusted hazard ratio.

HR_2_: Multi-variable adjusted hazard ratio.

Multi-variable adjusted hazard ratios are adjusted for age, smoking, body mass index, systolic blood pressure, fasting blood glucose, and total cholesterol in a multivariate Cox model when appropriate.

Risks included lifestyle risks (smoking, drinking, physical inactivity, betel quid chewing, prolonged sitting and sleep duration), biomarkers [high resting heart rate, proteinuria and low glomerular filtration rate (GFR), elevated C-reactive protein (CRP) or white blood cell (WBC), mild anemia, elevated Aspartate aminotransferase (AST), high uric acid, low fasting blood glucose, high and low total cholesterol, hepatitis B surface antigen carrier, low “high density cholesterol” (HDL), and high triglycerides] and chronic diseases [systolic or diastolic hypertension, pre-hypertension, diabetes, pre-diabetes, metabolic syndrome, obesity, underweight, COPD and restrictive lung disease].
