# Supplementary material for: Converting health risks into loss of life years - a paradigm shift in clinical risk communication
Source: Aging (Albany NY). 2021 Sep 7;13(17):21513–25. doi: 10.18632/aging.203491 (PMC8457574; doi:10.18632/aging.203491)
Supplement: Supplementary Table 2 [file aging-13-203491-s002.pdf]

SUPPLEMENTARY TABLE

Supplementary Table 2. Proportion of subjects with risk(s) losing 4 years or more in the cohort.

	Men	Women	Total
≥ 1 risk factor	88.7%	94.1%	91.5%
≥ 2 risk factors	63.7%	72.8%	68.4%
≥ 3 risk factors	38.5%	43.2%	40.9%
≥ 4 risk factors	20.6%	20.7%	20.6%
≥ 5 risk factors	9.8%	8.8%	9.2%
